# Determining the minimal important change of the recap of atopic eczema (RECAP) instrument in clinical trials

**DOI:** 10.1002/ski2.470

**Published:** 2024-10-26

**Authors:** Arabella Baker, Beth Stuart, Laura Howells, Eleanor J. Mitchell, Kim S. Thomas

**Affiliations:** ^1^ Centre of Evidence Based Dermatology School of Medicine University of Nottingham Nottingham UK; ^2^ College of Nursing and Midwifery Birmingham City University Birmingham UK; ^3^ Pragmatic Clinical Trials Unit Queen Mary University of London London UK; ^4^ Nottingham Clinical Trials Unit University of Nottingham Nottingham UK

## Abstract

**Background:**

The Recap of atopic eczema (RECAP) is a patient‐reported instrument designed to assess eczema control. There is a lack of evidence on the interpretability of change scores in clinical trials.

**Objectives:**

To calculate the smallest detectable change (SDC) in RECAP and estimate the minimal important change (MIC) for RECAP using various calculation methods in three eczema clinical trial datasets.

**Methods:**

In this study, four anchor‐based methods (within‐person score change, between‐patient score change, predictive modelling, receiver operating characteristic curve) and a distribution‐based method (effect size) was used to determine the MIC of RECAP. The trial datasets involved children (0–12 years), young people (13–25 years) and adults (>25 years) with all eczema severities.

**Results:**

A total of 698 participants were included in this study. The SDC was between 1.74 and 1.80. For the anchor‐based methods, the patient global assessment anchor provided MIC values ranging from 2.35 to 3.94 and the patient oriented eczema measure anchor yielded values between 1.11 and 3.62. The MIC for the distribution‐based method ranged from 2.66 to 3.06, respectively.

**Conclusions:**

The interpretability of RECAP was improved by establishing MIC values and the following thresholds are suggested for interpreting changes in RECAP scores: <2.0 points is possibly a measurement error; 2.0–2.9 points denotes a small improvement that may be clinically relevant; 3.0–3.9 points indicates an improvement that is likely to be clinically important and ≥4.0 points is highly likely to represent a clinically important change.

**What is already known?**

- Recap of atopic eczema (RECAP) is a patient‐reported instrument, recommended by the Harmonising Outcome Measures for Eczema (HOME) initiative for measuring the core outcome domain of eczema control in eczema clinical trials.
- Previous studies have demonstrated good validity, reliability and responsiveness.
- There is a lack of evidence on the interpretability of change scores in clinical trials.

**What does this study add?**

- This study calculated the minimal important change (MIC) of RECAP in three clinical trial datasets involving children, young people and adults.
- Varied methods and anchors were used to calculate the MIC.
- A change score of ≥2.0 points is likely to be considered clinically important with varying degrees of certainty.

## 1 INTRODUCTION

Atopic eczema (AE), also known as atopic dermatitis, is a common inflammatory skin condition with a high global prevalence, affecting 20% of children and 7%–14% of adults.^1^, ^2^, ^3^ AE is characterized by a relapsing‐remitting pattern, leading to sleep disturbance, reduced functioning and decreased health‐related quality of life.^4^, ^5^ Uncontrolled AE imposes a significant burden on both patients and healthcare services.^6^, ^7^ The Recap of atopic eczema (RECAP) patient‐reported instrument was developed to assess eczema control.^8^ RECAP is recommended by the Harmonising Outcome Measures for Eczema (HOME) initiative for measuring the long‐term control of AE in clinical trials.^9^

Previous studies have reported good validity, reliability, responsiveness and content validity of RECAP.^10^, ^11^, ^12^ Recently, two prospective studies in tertiary settings have evaluated the interpretability of RECAP in adults and children aged <12 years.^13^, ^14^ However, the interpretability of RECAP in clinical trials has not yet been assessed. The MIC is an essential aspect of interpretability, representing the smallest change in scores that is considered clinically significant.^15^, ^16^, ^17^ The MIC of RECAP denotes meaningful changes in AE control, helping to gauge treatment effectiveness and evaluate patient outcomes. Determining the MIC in clinical trials is crucial because it enhances our understanding of the instrument, providing more insights into its performance and potential utility for sample size calculations and for establishing statistical power in trials.^18^

The present study aimed to determine the interpretability of RECAP change scores in three clinical trial datasets and fill a validation gap for the HOME initiative by calculating the smallest detectable change (SDC) in RECAP, establishing the MIC of RECAP through the use of various calculation methods and comparing the MIC estimates provided by a single‐item anchor and a multi‐item anchor. Findings provide thresholds for the interpretation of RECAP change scores, helping to enhance the use of RECAP in research and clinical settings and ultimately advancing the understanding of AE control.

## 2 PATIENTS AND METHODS

### 2.1 Sources of data

Data from three online randomized controlled AE trials (trials A, B and C)^19^, ^20^ were used in this study (Table 1). All included trials measured AE control using the RECAP instrument, and eczema severity was also measured by the patient oriented eczema measure (POEM).^21^ (21) The global assessment of eczema severity was assessed in trial A only, through the Patient Global Assessment (PGA).

**TABLE 1:** Overview of trials included in this study.

Trial characteristics	Trial A (*n* = 296)	Trial B (*n* = 340)	Trial C (*n* = 337)
Design	Online RCT	Online RCT	Online RCT
Recruitment method	Social media	Primary care	Primary care
Participants			
Age (years)	2–74	0–12	13–25
Eczema severity, *n* (%)^a^			
Mild	36 (12%)	53 (16%)	21 (6%)
Moderate	135 (46%)	212 (62%)	184 (55%)
Severe	125 (42%)	75 (22)	132 (39%)
Intervention	Weekly symptom assessments	Online behavioural intervention	Online behavioural intervention
Control	No assessments	Usual care	Usual care
Timing of outcome assessments	Baseline week 8	Baseline week 24	Baseline week 24
Publication	Baker et al. (2023)	Santer et al. (2022)	Santer et al. (2022)
Participants included in MIC analysis	219	235	232

Abbreviation: RCT, randomised controlled trial.

^a^ Based on Patient Oriented Eczema Measure (POEM) scores.

### 2.2 Outcome measures

The main outcome of interest for this study was the RECAP instrument, which was developed and initially validated both in adults with eczema and parents of children with eczema (proxy). RECAP is a seven‐item instrument, capturing patient‐perceived eczema control over the last week.^8^ Each item carries equal weight and is rated between 0 and 4 points, providing a total score from 0 to 28 with higher scores indicating less eczema control.^8^

### 2.3 Anchors

The following two anchors were employed in this study: change scores in PGA (trial A) and change scores in POEM (trials A, B and C) as described in Table 2.

**TABLE 2:** Anchors used for calculating the MIC.

Outcome measure name (anchor)	Questions	Response options	Recall period
PGA (single‐item anchor)	1. How is your eczema today?	Clear Almost clear Mild Moderate Severe Very severe	On the day of assessment
POEM (multi‐item anchor)	Over the last week, on how many days has your skin been itchy because of your eczema? Over the last week, on how many nights has your sleep been disturbed because of your/their eczema? Over the last week, on how many days has your skin been bleeding because of your eczema? Over the last week, on how many days has your skin been weeping or oozing clear fluid because of your eczema? Over the last week, on how many days has your skin been cracked because of your eczema? Over the last week, on how many days has your skin been flaking off because of your eczema? Over the last week, on how many days has your skin felt dry or rough because of your eczema?	No days 1–2 days 3–4 days 5–6 days Every day	Previous week

Abbreviations: PGA, patient global assessment; POEM, patient oriented eczema measure.

Since the MIC anchor‐based methods contrast two prespecified groups, it is preferred to focus the analysis of change on one direction at a time (improvement or deterioration).^16^ Given that trials are usually looking at clinically significant improvements, this study exclusively focused on improvement and excluded participants whose eczema had deteriorated. The two groups used for the anchor‐based methods were the minimum important improvement and the no change groups.

In order to determine the suitability of the individual anchors, their linear relationship with the RECAP change scores was assessed using Pearsons' *r* correlation and a minimum correlation of *r* = 0.50 was required to deem the anchor suitable.^22^

#### 2.3.1 Patient global assessment (single‐item anchor)

PGA was selected as an anchor due to its simplicity, common use in MIC calculations and widespread recognition as a meaningful anchor.^23^ PGA assesses global eczema severity and scores range from 0 (clear) to 5 (very severe), where higher scores represent more eczema severity (Table 2). To obtain a scale where higher scores represent less severe eczema, scores were reversed and positive change scores represented improvement. To provide a single item anchor, PGA scores were converted into a change score by subtracting the score at baseline from the score at week 8. The PGA change scores ranged from 0 (no change) to 1 (minimum important improvement) where a positive 1 change on the PGA indicated a meaningful improvement, denoting a change in severity banding.

#### 2.3.2 Patient oriented eczema measure (multi‐item anchor)

POEM was chosen as an anchor to be able to include all three trials (A, B, C) in this study and also to explore the usefulness of using POEM as an anchor in MIC studies. POEM is a seven item instrument that measures eczema severity and scores range from 0 (clear) to 28 (very severe eczema), a decrease in scores denotes less eczema severity (Table 2).^21^ Similar to the PGA anchor, POEM scores were reversed so that positive change scores represented improvement. POEM has an established value of ≥3.0 points.^23^, ^24^ Whereas, the SDC, that is, a change beyond measurement error is reported as 2.0 points.^24^ Considering these existing values, POEM change scores were pre‐specified as POEM +1, 0, −1 point change (no change) and 3.0 point change (minimum important improvement). These categories ensured the inclusion of those who had a meaningful change in their POEM scores.

### 2.4 Statistical analysis

This study included participants from trial A who were aged ≥14 years and had available paired measurements at baseline and week 8 for PGA, POEM and RECAP. Trial participants younger than 14 years of age were not included in this study due to the very small sample size (*n* = 15). Additionally, participants from trials B and C were included if they had completed paired measurements at baseline and week 24 for RECAP and POEM. Prior to conducting the analyses, change scores were calculated for each instrument. The inclusion of 686 participants from the three trials was considered sufficient, exceeding the recommended minimum of 100 participants.^25^ Analyses were conducted in Stata statistical software, version 17.0.^26^ All analyses are reported descriptively. MIC calculations were conducted using five different methods to explore the variation in MIC estimates produced by each method.

#### 2.4.1 Smallest detectable change (SDC)

The SDC was derived, using the following formula^16^:

$$\text{SDC}=1.96\times \sqrt{2}\times \text{standard}\;\text{error}\;\text{of}\;\text{measurement}\;\left(\text{SEM}\right)$$

The SEM was calculated as follows:

$$\text{SEM}={\text{SD}}_{\text{pooled}}\times \sqrt{1-\mathrm{ICC}}$$

The following formula was used for SD_pooled_:

$${\text{SD}}_{\text{pooled}}=\sqrt{\frac{{SD}_{1}^{2}+{SD}_{2}^{2}}{2}}$$

The intraclass correlation coefficient (ICC) _agreement_ score was derived from the test‐retest reliability of RECAP.^13^ To determine the appropriateness of the ICC used for calculating the SDC, we assessed the similarity in baseline heterogeneity comparing the SDs across the trial datasets. Following guidance,^16^ this approach was considered acceptable as there was similar baseline heterogeneity (SD) in the trial datasets (trial A SD_baseline_ = 6.12; trial B SD_baseline:_ 5.32; trial C SD_baseline_: 5.37) versus the dataset used to perform the test–retest of RECAP (SD_baseline_: 8.0).

### 2.5 Anchor‐based methods

#### 2.5.1 Within‐patient score change

The MIC was determined by calculating the mean change in RECAP scores for the smallest improvement group on the relevant anchor.^22^

#### 2.5.2 Between‐patient score change

The MIC was estimated by using the mean difference in RECAP change scores between the mean change score of the smallest improvement group and the stable group as identified by the relevant anchor.^27^

#### 2.5.3 Predictive modelling method

This method uses logistic regression analysis with dichotomous outcomes to predict whether a participant belongs to the improved or the not improved group.^28^ The change in RECAP scores served as the primary predictor, whereas the improvement in the respective anchor was the dependent variable. The predictive modelling method is more precise than other anchor‐based methods, thus it is a preferred MIC calculation method.

The MIC was estimated using the following formula: ${\text{MIC}}_{\text{pred}}=\frac{\text{In}\left({\text{odds}}_{\text{pre}}\right)-C}{B}$

Here, *C* denotes the intercept, *B* is the correlation coefficient of the RECAP change score from the logistic regression model and $\text{In}\left({\text{odds}}_{\text{pre}}\right)=\frac{\text{proportion}\;\text{improved}\;\text{on}\;\text{the}\;\text{anchor}}{1-\text{proportion}\;\text{improved}\;\text{on}\;\text{the}\;\text{anchor}}$

It has been shown that the proportion of improved participants on the anchor can impact the estimation of the MIC.^29^ Specifically, when the proportion of improved participants is <50% the MIC can become a biased estimate. Therefore, adjusting for this proportion is necessary to ensure that it reflects the genuine MIC in a given sample. Since in all three trials the proportion of improved participants was <50% on the POEM anchor (trial A 46%; trial B 49%, trial C 45%) and 37% on the PGA anchor, the adjusted MIC was calculated.^29^

#### 2.5.4 Receiver operating characteristic (ROC) curve

The area under the curve of the ROC curve analysis was used to obtain the optimal cut‐off point for the RECAP change scores. This cut‐off point serves as a discriminating factor between the smallest improvement group and the stable group. The optimal ROC cut‐off point denotes the MIC of RECAP, which maximizes the Youden's J statistic of sensitivity‐(1‐specificity).^27^

### 2.6 Distribution‐based methods

#### 2.6.1 Effect size

This distribution‐based method is a measure of variability, whereby the variation among a group of scores is assessed. This approach solely relies on the distribution of baseline RECAP scores without relating it to an anchor for assessing the degree of change. The value of 0.5 standard deviation (SD) of baseline RECAP scores corresponds to the MIC.^30^ To estimate the MIC, the baseline SD of baseline RECAP scores was calculated in each trial.

## 3 RESULTS

**FIGURE 1:**
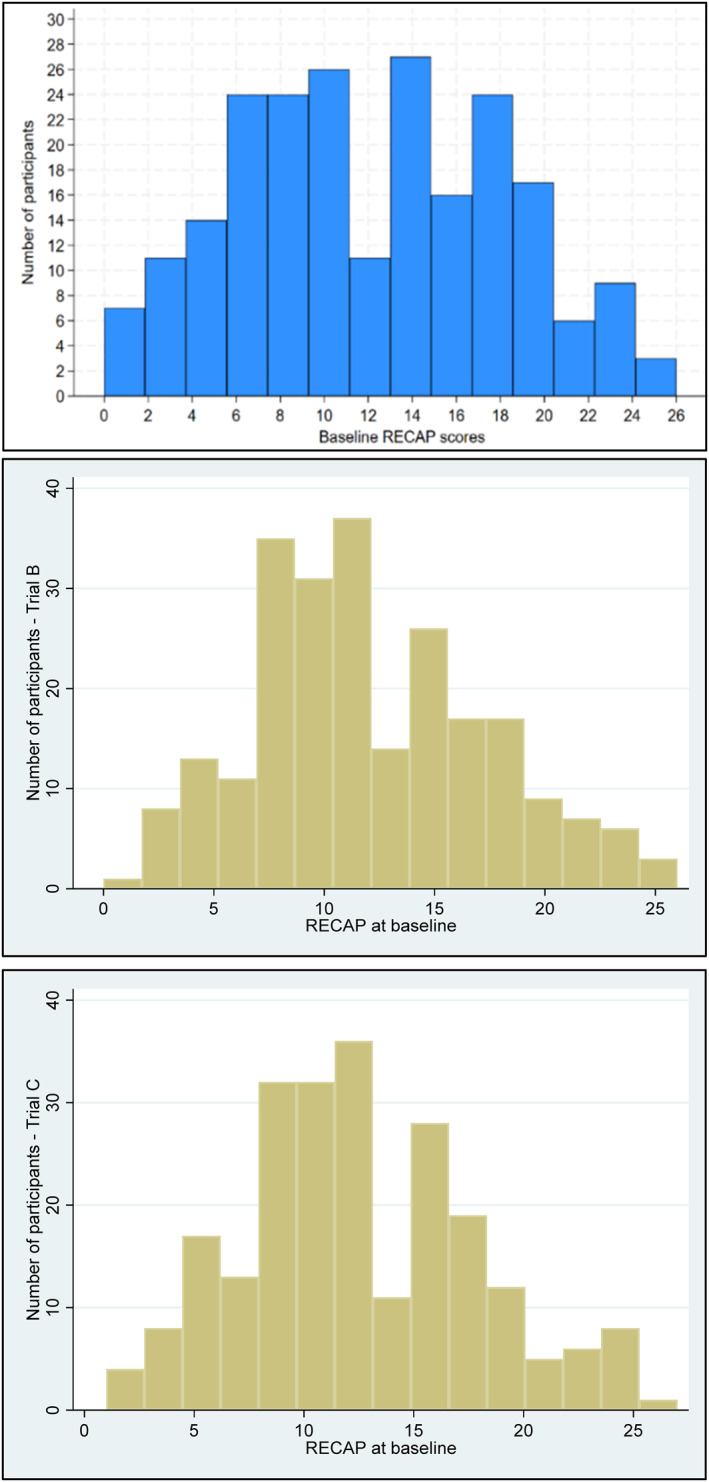
Distribution of baseline RECAP scores. RECAP, recap of atopic eczema.

A total of 686 participants were included in this study (trial A *n* = 219; trial B *n* = 235; trial C *n* = 232). The demographic and clinical characteristics of included participants is shown in Table 3. The distribution of baseline RECAP scores according to individual trials is illustrated in Figure 1.

**TABLE 3:** Baseline characteristics of included participants.

Participant characteristics	Trial A *n* = 219	Trial B *n* = 235	Trial C *n* = 232
Age (years)			
Mean (SD)	28.48 (14.14)	4.68 (3.35)	19.1 (3.38)
Minimum, maximum	14–74	0–12	13–25
Gender, *n* (%)			
Male	52 (23.7)	116 (49.6)	49 (21.1)
Female	162 (74.0)	118 (50.4)	183 (78.9)
Other	1 (0.5)		
Prefer not to say	4 (1.8)		
Ethnicity, *n* (%)			
White	167 (76.3)	202 (86.3)	197 (86.0)
Asian or Asian British	28 (12.8)	14 (6.0)	16 (7.0)
Black, African, Black British or Caribbean	10 (4.6)	5 (2.1)	10 (4.4)
Mixed or multiple ethnic groups	11 (5.0)	8 (3.4)	2 (0.9)
Another ethnic group	3 (1.3)	5 (2.1)	4 (1.8)
Baseline RECAP, mean (SD)	12.0 (6.12)	12.1 (5.32)	12.4 (5.37)
RECAP change scores	1.4 (5.6)	2.7 (5.84)	1.5 (5.85)

Abbreviations: PGA, patient global assessment; POEM, patient oriented eczema measure; RECAP, recap of atopic eczema; SD, standard deviation.

### 3.1 Calculating the SDC

The ICC was 0.988, leading to the SDC being 1.80 points in trial A and 1.74 points in both trial B and trial C.

### 3.2 Calculating the MIC

#### 3.2.1 Anchor‐based methods

The correlation between the PGA anchor and RECAP change scores was *r* = 0.62, indicating its suitability as an anchor in this study. Similarly, the POEM anchor was also deemed appropriate, demonstrating correlations of *r* = 0.68 (trial A), *r* = 0.74 (trial B) and *r* = 0.68 (trial C).

#### 3.2.2 Within‐person score change

The PGA anchor yielded a MIC value of 3.94 and the POEM anchor provided MIC estimates ranging from 1.45 to 3.13. Table 4 demonstrates all the MIC values obtained in this study.

**TABLE 4:** Summary of MIC values provided by the PGA and POEM anchors for each trial.

Calculation method	PGA anchor MIC values	POEM anchor MIC values	POEM anchor MIC values	POEM anchor MIC values
	Trial A ≥14 years old	Trial A ≥14 years old	Trial B 0–12 years old	Trial C 13–25 years old
Anchor‐based methods				
Within‐person change score	3.94	2.33	3.13	1.45
Between‐person change score	3.52	2.06	3.61	1.11
Predictive modelling	4.36	2.08	2.91	2.5
Adjusted predictive modelling	2.35	1.77	2.81	1.84
ROC method	0.5	0.5	2.5	0.5
Distribution‐based method				
Effect size	3.06	3.06	2.66	2.69

Abbreviations: MIC, minimum important change; PGA, patient global assessment; POEM, patient oriented eczema measure; ROC, receiver operating characteristic.

#### 3.2.3 Between‐person score change

This method produced MIC estimates of 3.52 for the PGA anchor and 1.11–3.61 for the POEM anchor.

#### 3.2.4 Predictive modelling method

In trial A, the PGA anchor provided a MIC estimate of 4.36 and after adjusting by baseline disease severity the MIC estimate was 2.35. Whereas the POEM anchor yielded MIC values of 2.08–2.91 and results for the adjusted MIC were between 1.77 and 2.81, respectively.

#### 3.2.5 ROC curve method

The MIC using the PGA anchor was 0.5 and the MIC for the POEM anchor ranged between 0.5 and 2.5.

### 3.3 Distribution‐based method

#### 3.3.1 Effect size

SD of baseline RECAP scores was 6.12 (trial A), 5.32 (trial B) and 5.37 (trial C). Using the 0.5 SD of baseline scores resulted in a MIC of 3.06. trial B yielded a MIC of 2.66 and the MIC was 2.69 in trial C.

## 4 DISCUSSION

This study progresses our understanding of how to interpret RECAP change scores in clinical trials. The MIC estimates observed in the three datasets ranged from 1.11 (between patient change score method) and 4.36 (unadjusted predictive modelling method). The choice of calculation method impacts on the MIC estimates; thus, due consideration should be given to the interpretation of published MIC values. Notably, there is an ongoing debate on the most appropriate and optimal MIC calculation methods, posing a challenge in the selection of approaches.^31^ Anchor‐based methods are preferred due to explicitly measuring the importance of the change and providing more theoretically sound estimations compared to the distribution‐based methods.^32^ Amongst anchor‐based approaches, the calculation methods are gradually evolving and becoming more advanced. Initially, this was evident with the emergence of the ROC method for calculating the MIC followed by a more recent development of the predictive modelling method. The latter method is favoured by many, including the Consensus‐Based Standards for the Selection of Health Measurement Instruments (COSMIN) group because it provides greater precision than the ROC method, and it is also superior to other anchor‐based methods.^32^ Whereas, the distribution‐based methods are practical and provide statistical thresholds for the margins of error. Findings of this study support the views of Turner and colleagues^33^ that 0.5 SD is a good approximation of the MIC.

This study adhered to current recommendations, involving multiple independent anchors and calculation methods in different datasets followed by the triangulation of results that allowed to gain a more comprehensive interpretation and understanding of the MIC of RECAP.^22^, ^27^, ^32^ The PGA and POEM anchors provided a range of MIC estimates for the different calculation methods. In general, the PGA yielded higher MIC values between 3.52 and 4.36 while the POEM anchor produced values ranging from 1.11 to 3.61. This inconsistency in estimates was likely related to the fact that both single‐item and multi‐item anchors were used in this study, which required to define the smallest improvement groups slightly differently (PGA = 1.0, POEM = 3.0). Consequently, a change of 3.0 points on POEM is possibly a smaller change than a change of 1.0 points on the PGA, leading to smaller MIC values. Furthermore, the different sample sizes in the predefined groups on the two anchors may have caused further variability. For instance in trial A, there were 45 participants in the stable group for POEM, compared to 96 participants who remained stable on the PGA suggesting that the PGA is less sensitive to change. Additionally, the ROC method yielded low MIC values of 0.5 in trials A and B. Since this value falls below the SDC, these values were excluded.

Overall, the results of this study demonstrated that the MIC is not a fixed value and a single undisputed MIC estimate is not advisable. MIC is a variable concept and its value depends on different factors, including choice of anchor, calculation methods, disease severity, type of intervention and setting, resulting in varied MIC estimates.^18^, ^34^ The findings of this study have been used to generate broad recommendations on how one might interpret changes in RECAP scores as illustrated in Table 5. Given that MIC values were consistent across the three trials that included diverse age groups of participants, the proposed MIC values are likely to be appropriate across all age ranges.

**TABLE 5:** A guide for enhancing interpretation of change in RECAP scores.

Change in RECAP score	Suggested interpretation
0–1.9 points	Likely to be measurement error
2.0–2.9 points	Small improvement, that may be clinically important
3–3.9 points	Improvement that is likely to be clinically important
≥4 points	Improvement that is very likely to be clinically important

Abbreviation: RECAP, recap of atopic eczema.

Our results are broadly consistent with the findings of a previous study that suggested a MIC value of ≥4.0 points.^12^ However, the results are not directly comparable because the studies were conducted in different populations (self‐referral via community advertising for trial A); primary care for trials B and C and secondary care for the previous study). Further, the anchors used to assess change in the three trials differed from those used in the previous study and were measured at different timepoints. Therefore, decisions around the most appropriate MIC to use for a particular purpose is best tailored to the individual circumstances of the trial and intervention being evaluated.

A strength of this study is that it estimated the MIC of RECAP in accordance with best practice and COSMIN guidelines, using a range of calculation methods and multiple anchors.^25^ The inclusion of all disease severities and ages as well as the large sample size from three trial datasets further enhanced the robustness of the results.

The anchors in this study measured change from baseline to follow‐up. This approach is more advantageous than using retrospective measures of change, where participants are asked to reflect how their eczema has changed over a period of time. Retrospective self‐reports may be prone to recall bias, typically reflecting present state rather than baseline state.^22^, ^35^, ^36^

A limitation is that the choice of anchors was dictated by the existing trial datasets, which may not be ideal for conceptualising the MIC. The anchors did not assess the importance of change from the patient perspective, which is a recurring criticism of the anchors usually used for calculating MIC values.^32^ Additionally, some may argue that the anchors used in this study measured eczema severity rather than control, but these constructs are closely related. Eczema severity often indicates the frequency and intensity of symptoms, which inherently influences eczema control. Effective eczema control typically leads to reduced disease severity, highlighting that severity‐based anchors reflect critical elements of control and can provide valuable insights. Moreover, using a 1.0 point improvement on the PGA anchor to denote meaningful improvement may not entirely reflect the change that patients consider as important.

Since RECAP is a relatively new instrument, further research in various populations and settings is needed to help assess its performance in different scenarios. For instance, the SDC in RECAP has not yet been calculated, hence it was important to include it in this study to start establishing potential values. Furthermore, the ICC has not been previously determined in RCTs which is a necessary step for computing the SDC. The available trial datasets did not allow for calculating the test–retest reliability of RECAP, so we used the ICC from a prospective study instead.^13^ In order to improve the accuracy of the ICC estimate, further research on the test‐retest reliability of RECAP would be valuable.

## 5 CONCLUSIONS

This study has enhanced the interpretability of RECAP by employing multiple methods to calculate the MIC. Our results suggest that a change score of ≥2.0 points is likely to be considered clinically important with varying degrees of certainty. These results provide valuable insights for users of the instrument, aiding sample size calculation, interpretation of trial results and clinical significance. MIC values of an instrument facilitate evidence‐based decision‐making and support the integration of RECAP into clinical trials.

## CONFLICT OF INTEREST STATEMENT

Laura Howells and Kim S Thomas were involved in the original development of the Recap of atopic eczema (RECAP) questionnaire. They are members of the Harmonising Outcome Measures for Eczema (HOME) Executive Group.

## AUTHOR CONTRIBUTIONS

**Arabella Baker**: Conceptualization (equal); formal analysis (equal); methodology (equal); writing—original draft (lead). **Beth Stuart**: Conceptualization (equal); formal analysis (equal); methodology (equal); writing—review and editing (equal). **Laura Howells**: Conceptualization (equal); methodology (equal); writing—review and editing (equal). **Eleanor J. Mitchell**: Conceptualization (equal); methodology (equal); supervision (equal); writing—review and editing (equal). **Kim S. Thomas**: Conceptualization (equal); methodology (equal); supervision (equal); writing—original draft (equal).

## ETHICS STATEMENT

Ethical approval to conduct the trials used in this study were obtained from the University of Nottingham Research Ethics Committee (239‐0421) and from South Central‐Oxford A Research Ethics Committee (19/SC/0351). Participants provided permission for the reuse of deidentified data in secondary studies.

## PATIENT CONSENT

Not applicable.

## Data Availability

The data underlying this article will be shared on reasonable request to the corresponding author.
